# Traité De Médecine Légale

**Published:** 1848-09

**Authors:** 


### BIBLIOGRAPHICAL NOTICES.

*Traité de Médecine Légale*, par M. ORFILA, Doyen et Professeur de la Faculté de Médecine de Paris, &c. 4ème édit., revue, Corrigée et considerablement augmentée, Contenant en entier le Traité des Exhumations Juridiques par MM. Orfila et Lesueur avec Planches. 4 tom. 8vo. Paris, 1848.

This is a new edition of one of those admirable works, which have given to the name of Orfila a wide spread reputation. As a medico-legal writer he may be regarded as one of the chief contributors to our knowledge of what is demanded of the medical practitioner in courts of justice; whilst his experiments and observations on poisons have tended, more perhaps than those of any other observer, to elucidate a subject full of importance and difficulty. In his own adopted country, as elsewhere, he has been honoured as every contributor to science, and to the science of man especially, ought to be honoured; until recently, when the zeal—too often hasty, intemperate, and inconsiderate—which political changes engender—has led to his removal from the position in the Faculty of Medicine, which he had occupied so honourably and so ably, and the appointment of one in his place, whose political sentiments are more in accordance with those of "the powers that be." Alas! that any political considerations should ever be permitted to have weight in the realm of science.

The removal of such an individual is no degradation to him, whatever it may be to those by whose acts it has been effected; and in the revolution of sentiments that may yet occur, his star may even shine brighter and more gloriously than it has hitherto.

It would be as impracticable as it would be deemed unnecessary, to attempt in our pages anything like an analysis of these large volumes, occupying the space of nearly *two thousand six hundred pages*. They comprise a full dissertation on the views of M. Orfila and of French observers more especially, of the subjects embraced by them. It is to be regretted here—as in the case of so many of the French writers on Medicine, as well as on almost all subjects—that their attention is rarely directed to fellow-labourers elsewhere;—a circumstance which is apt to lead the superficial to believe, that all science is Gallic in its origin and extension; and we think that such has been the impression made upon not a few of the young physicians, who have listened to the expositions of the teachers in the French metropolis; whilst no such impression has been made upon those whose minds have been better prepared for observing and judging, by a lustre or two spent in the active study and exercise of their profession at home. When, moreover, a rare reference is made by French writers to English or American observers, the names are apt to be *estropiés* in such sort as to be scarcely recognizable, and the translation so perverted from the original, as to give an erroneous idea of the views of the writer.

Under the head of signs of pregnancy, (vol. 1, p. 208,) we have an ample notice of the value of kyestein as a diagnostic mark; and of the researches of "MM. Nauches, [Nauche,] Eguisier and Tanchou, in France; of Doctors Letheby and Stark in England, and of Kant [?] in America." p. 229. This "Kant" is Dr. E. K.

Kane of this city, to whose excellent paper—the result of accurate and sustained observation—we have more than once referred in the pages of this journal.

At the end of each subject, M. Orfila gives reports, drawn up by authority. One of these on Pregnancy we extract, in order to show the mode in which they are required to be made by the French courts.

"*First Report.*—We, the undersigned, a Doctor of Medicine of the Faculty of Strasburg, on the requisition of the Procureur du Roi, signified to us by the Sieur D. officers, proceeded to say:—The 12th of June, at noon, accompanied by M. V., commissary of police, to the prison in which Madame * * * aged twenty-five years, is confined, to discover whether she is *enceinte.* On entering the chamber No. 2, we found the said lady, who affirmed to us, that she was six months gone with child, as indicated by the aversion for food, and vomiting, which she had experienced for that time, the suppression of the catamenia, gradual tumefaction of the abdomen, and especially by the movements which she had felt for two months in the abdomen.

"Madame B. being in the erect attitude, the index finger of the right hand was introduced into the vagina, whilst the left hand was kept applied upon the abdomen; this permitted us to discover, that the neck of the uterus was drawn upwards and backwards: that the fundus of the organ, perfectly developed, corresponded with the umbilicus, and that the movements of *ballottement* could be detected. The urine contained *kyestein.* With the aid of the stethoscope, placed over the space which separates the umbilicus from the crural arch, we heard at least one hundred and thirty double pulsations per minute; and over another part of the abdomen, with the same instruments, simple pulsations, isochronous with the pulse of the mother, could be distinguished.

"These facts lead us to conclude, that Madame * * * is about six months advanced in pregnancy. In testimony whereof, &c. Paris, June 10th, 1820."

The present edition contains the entire treatise on Juridical Exhumations by MM. Orfila and Lesueur, which occupies upwards of three hundred pages of the first volume. It is an elaborate inquiry into the changes produced on the dead body at different periods after sepulture, and under varied circumstances; and is full of interest to the medico-legist.

The whole work ought, indeed, to hold a conspicuous place in the library of those who are desirous—and who are not?—of maintaining themselves on a level with the existing condition of the science.

